# PyOrthoANI, PyFastANI, and Pyskani: a suite of Python libraries for computation of average nucleotide identity

**DOI:** 10.1093/nargab/lqaf095

**Published:** 2025-07-11

**Authors:** Martin Larralde, Georg Zeller, Laura M Carroll

**Affiliations:** Structural and Computational Biology Unit, EMBL, 69117 Heidelberg, Germany; Leiden University Center for Infectious Diseases (LUCID), Leiden University Medical Center, 2333ZA Leiden, Netherlands; Structural and Computational Biology Unit, EMBL, 69117 Heidelberg, Germany; Leiden University Center for Infectious Diseases (LUCID), Leiden University Medical Center, 2333ZA Leiden, Netherlands; Center for Microbiome Analyses and Therapeutics, Leiden University Medical Center, 2333ZA Leide, Netherlands; Department of Clinical Microbiology, SciLifeLab, Umeå University, 90187 Umeå, Sweden; Laboratory for Molecular Infection Medicine Sweden (MIMS), Umeå University, 90187 Umeå, Sweden; Umeå Centre for Microbial Research (UCMR), Umeå University, 90187 Umeå, Sweden; Integrated Science Lab (IceLab), Umeå University, 90187 Umeå, Sweden

## Abstract

The average nucleotide identity (ANI) metric has become the gold standard for prokaryotic species delineation in the genomics era. The most popular ANI algorithms are available as command-line tools and/or web applications, making it inconvenient to incorporate them into bioinformatic workflows, which utilize the popular Python programming language. Here, we present PyOrthoANI, PyFastANI, and Pyskani, Python libraries for three popular ANI computation methods. ANI values produced by PyOrthoANI, PyFastANI, and Pyskani are virtually identical to those produced by OrthoANI, FastANI, and skani, respectively (adjusted *R*^2^*>*0.999). Compared to OrthoANI, PyOrthoANI is, on average, 3× faster per genome, while PyFastANI has multithreading support for single queries. All three libraries integrate seamlessly with BioPython, making it easy and convenient to use, compare, and benchmark popular ANI algorithms within Python-based bioinformatic workflows, software programs, and notebooks. Each library is available as part of the Python Package Index repository under the open-source MIT license, with source code available via GitHub (PyOrthoANI, https://github.com/althonos/orthoani; PyFastANI, https://github.com/althonos/pyfastani; Pyskani, https://github.com/althonos/pyskani).

## Introduction

The average nucleotide identity (ANI) metric of genomic similarity is arguably the most popular method for prokaryotic species delineation in the genomics era [1, 2]. The calculation of ANI values shared between two genomes is a crucial step in many bioinformatic pipelines, including popular methods/workflows for prokaryotic species identification [3, 4], within-species lineage/strain delineation [5, 6], and general prokaryotic (meta)genomic data analysis [7, 8].

While numerous ANI algorithm implementations have been developed, nucleotide BLAST-based ANI (ANIb) algorithms are considered to be the gold standard [1, 9]. ANIb algorithms are accurate in the sense that they share a strong correlation with experimentally determined DNA–DNA hybridization values [2, 10–12]. However, due to the high time complexity of BLAST and similar alignment-based algorithms, ANIb algorithms are notoriously slow [1] and thus most appropriate for users with smaller datasets (e.g. up to ≈10^3^ genomes/10^6^ pairwise comparisons), who prioritize accuracy over speed.

To overcome the computational limitations of ANIb, alignment-free ANI algorithms have been developed, most notably FastANI [1] and skani [9]. Both FastANI and skani forgo some accuracy in favor of speed (i.e. they produce ANI values, which correlate with, but are not necessarily equivalent to, ANIb), and as such, they can readily scale to massive genomic datasets (e.g. ≥10^4^ genomes/10^8^ pairwise comparisons) [1, 9]. However, identifying the optimal alignment-free ANI algorithm for a given dataset is not always straightforward. FastANI is ≥50× faster than ANIb methods and is more accurate than skani on reference-quality genomes [1, 9]. skani, on the other hand, is >20× faster than FastANI and is more accurate on fragmented, incomplete metagenome-assembled genomes (MAGs) [9]. Thus, in addition to considering dataset size and algorithm speed–accuracy trade-off, users may want to consider dataset composition (e.g. isolate genomes versus MAGs) and quality when selecting the optimal ANI algorithm for their dataset.

Regardless of whether they prioritize accuracy or speed, the most popular ANI algorithms/methods [e.g. FastANI, skani, ANI by Orthology (OrthoANI), JSpeciesWS, PyANI] are available as command-line tools and/or web applications [1, 9, 12–14]. This makes it inconvenient for bioinformaticians to incorporate ANI algorithms into bioinformatic workflows, which utilize the popular Python programming language [15].

Here, we present a suite of Python libraries for popular ANI algorithms, specifically (i) PyOrthoANI, a Python-based implementation of the OrthoANI algorithm (a highly accurate ANIb method) [12]; (ii) PyFastANI, and (iii) Pyskani, Python bindings for the FastANI and skani algorithms, respectively (fast, alignment-free methods) [1, 9]. Each Python library integrates seamlessly with BioPython [16], making it simple and convenient to perform ANI computations within Python-based bioinformatic workflows, software programs, and notebooks (e.g. Jupyter) [17]. By providing a unified Python interface, our suite allows users to easily swap out different ANI algorithms, making it simple and convenient to test, compare, and benchmark methods.

## Materials and methods

The PyOrthoANI algorithm (https://github.com/althonos/orthoani) was implemented in the same manner as the original OrthoANI Java implementation [12]. Briefly, to calculate ANI values between a query and reference genome, both genomes are partitioned into 1020-bp-long fragments. Fragments that are <1020 bp in length and/or contain >80% ambiguous (*N*) nucleotides are discarded. Nucleotide BLAST (blastn) [18] values are then calculated between the set of query and reference genome fragments using the following blastn parameters (all other parameters are set to their respective defaults): -task blastn, -evalue 1e-15, -xdrop_gap 150, -dust no, -penalty -1, -reward 1, -num_alignments 1, -outfmt 7. The resulting fragments are considered to be orthologous if they produce reciprocal best hits, which cover at least 35% of the total length of the fragment. Final ANI values are calculated by averaging the nucleotide identity values for all reciprocal blastn hits.

For PyFastANI (https://github.com/althonos/pyfastani), the original FastANI code (written in C++) [1] was wrapped into a Python extension module using the Cython language (v3.0) [19]. While PyFastANI uses the original FastANI code for hashing and core-genome identity computations, we reimplemented the sketching to support passing plain Python strings as input sequences. In addition, we implemented serialization/deserialization support to allow querying a reference database several times. To speed up the querying of individual sequences, we parallelized the fragment sketching step using Python thread pools and re-entrant code.

For Pyskani (https://github.com/althonos/pyskani), the original skani code (written in Rust) [9] was wrapped into a Python extension module using the PyO3 library (v0.22.5; https://pyo3.rs) for bindings generation. To accelerate querying, we implemented a more generic strategy for the storage of reference markers, allowing to either load the markers from a file iteratively (as in the original skani) or pre-load them in memory to reduce I/O costs for successive querying.

Validation and benchmarking were carried out on the five (meta)genomic datasets used to validate and benchmark FastANI [*n*= 14 952 total (meta)genomes]: (i) Dataset 1 (D1), with 1662 closed prokaryotic genomes from NCBI’s RefSeq database; (ii) Dataset 2 (D2), with 571 draft genomes derived from *Bacillus cereus* group isolates; (iii) Dataset 3 (D3), with 4350 draft genomes derived from *Escherichia coli* isolates; (iv) Dataset 4 (D4), with 468 draft genomes derived from *Bacillus anthracis* isolates; and (v) Dataset 5 (D5), with 7901 MAGs derived from public metagenomes (Supplementary Text) [1, 20]. Each of the following methods was used to calculate ANI values between each (meta)genome in Datasets D1–D5 and its respective query genome (Supplementary Text): (i) OrthoANI (OAT_cmd.jar v1.40) [12]; (ii) FastANI v1.33 [1]; (iii) skani v0.1.4 [9]; (iv) PyOrthoANI v0.6.0 (developed here); (v) PyFastANI v0.6.0 (developed here); and (vi) Pyskani v0.1.2 (developed here). Five methods (all but Pyskani) were evaluated using 1, 8, and 16 CPUs in triplicate; Pyskani was evaluated using 1 CPU in triplicate, as skani, and thus, Pyskani, does not parallelize when performing a single pairwise distance computation (per the skani source code and as demonstrated here, Supplementary Fig. S1 and Supplementary Text; *n*= 717 651 total ANI computations). For each computation, “trace” in Nextflow v24.04.2 [21] was used to log speed (real/wall clock time) and memory usage [peak resident set size (RSS); Supplementary Text].

## Results and discussion

**Figure 1.**
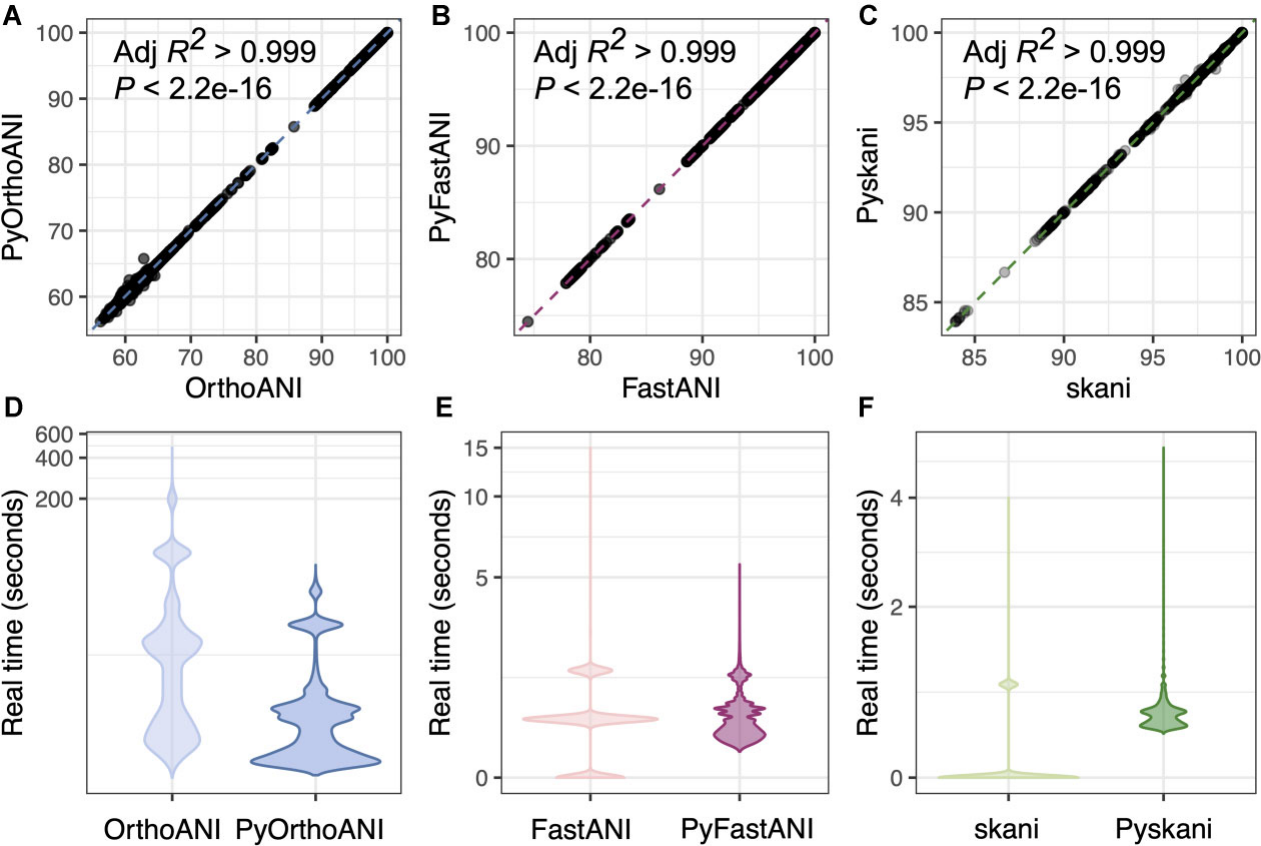
Correlation between ANI values produced by (**A**) PyOrthoANI, (**B**) PyFastANI, and (**C**) Pyskani (*Y*-axes) with ANI values produced by OrthoANI, FastANI, and skani, respectively (*X*-axes) for genomes ubsed in the FastANI validation/benchmarking datasets (black dots; Supplementary Tables S1–S6). Dashed lines denote the best-fitting linear model for each method pair, with adjusted *R*^2^ and *P*-values reported in the upper left corner of each subplot. Pyskani values were multiplied by 100. Per-genome real (wall clock) time in seconds (Y-axes, log-scale) for(**D**) OrthoANI/PyOrthoANI, (**E**) FastANI/PyFastANI, and (**F**) skani/Pyskani ((X-axes), using 1, 8, and/or 16 CPUs on the FastANI validation/benchmarking datasets (violin plots; Supplementary Table S7). For fairness, PyFastANI and Pyskani times include the time it took to load Python modules and parse genomes using BioPython (performed for every genome/computation). For extended versions of this figure, see Supplementary Figs S1–S7. Raw data used to construct all plots are available in Supplementary Tables S1–S7.

Using each of the five datasets used to validate and benchmark FastANI [*n*= 14 952 total (meta)genomes] [1], we compared ANI values produced by PyOrthoANI, PyFastANI, and Pyskani to those produced by OrthoANI, FastANI, and skani, respectively. We additionally benchmarked the speed of all six methods on each genome individually using 1, 8, and/or 16 CPUs in triplicate (*n*= 717 651 total ANI computations; Fig. 1, Supplementary Figs S1–S7, Supplementary Tables S1–S7, Supplementary Text).

ANI values calculated by PyOrthoANI, PyFastANI, and Pyskani were virtually identical to those produced by OrthoANI, FastANI, and skani, respectively (adjusted *R*^2^> 0.999 and *P*< 2.2e−16 for all methods; Fig. 1A–C). Compared to OrthoANI, PyOrthoANI was, on average, 3× faster per genome (Fig. 1D). PyFastANI and Pyskani performed similarly to FastANI and skani, respectively, even when Python module load times and genome parsing (via BioPython) were included in the PyFastANI/Pyskani runtime; however, differences in FastANI/PyFastANI and skani/Pyskani runtime and memory usage varied by dataset (Fig. 1E and F, Supplementary Figs S1–S7).

Overall, PyOrthoANI, PyFastANI, and Pyskani enable users to perform ANI computations within Python-based software, workflows, and notebooks. Because each Python library integrates with BioPython and is easily interchangeable, we anticipate that our Python suite will be particularly useful for comparing/benchmarking ANI algorithms, and for developers/users who frequently encounter highly heterogeneous datasets (e.g. genomic datasets varying in size, quality, and isolate/MAG composition) that require flexibility in ANI computation algorithms.

## Supplementary Material

lqaf095_Supplemental_Files

## Data Availability

PyOrthoANI, PyFastANI, and Pyskani are available (i) as part of the Python Package Index repository under the open-source MIT license at https://pypi.org/project/orthoani/, https://pypi.org/project/pyfastani/, and https://pypi.org/project/pyskani/, respectively; (ii) via GitHub (source code) at https://github.com/althonos/orthoani, https://github.com/althonos/pyfastani, and https://github.com/althonos/pyskani, respectively; and (iii) as Singularity containers (used for benchmarking) at https://cloud.sylabs.io/library/lmc297/pyorthoani/pyorthoani, https://cloud.sylabs.io/library/lmc297/pyfastani/pyfastani, and https://cloud.sylabs.io/library/lmc297/pyskani/pyskani, respectively. Source code is additionally available as Supplementary data.
