# Supplementary material for: PyOrthoANI, PyFastANI, and Pyskani: a suite of Python libraries for computation of average nucleotide identity
Source: NAR Genom Bioinform. 2025 Jul 11;7(3):lqaf095. doi: 10.1093/nargab/lqaf095 (PMC12246781; doi:10.1093/nargab/lqaf095)
Supplement: lqaf095_Supplemental_Files [file lqaf095_supplemental_files.zip › supplementary_material.tar.gz!/supplementary_material/SuppFigS2_full_violin_v2.pdf]

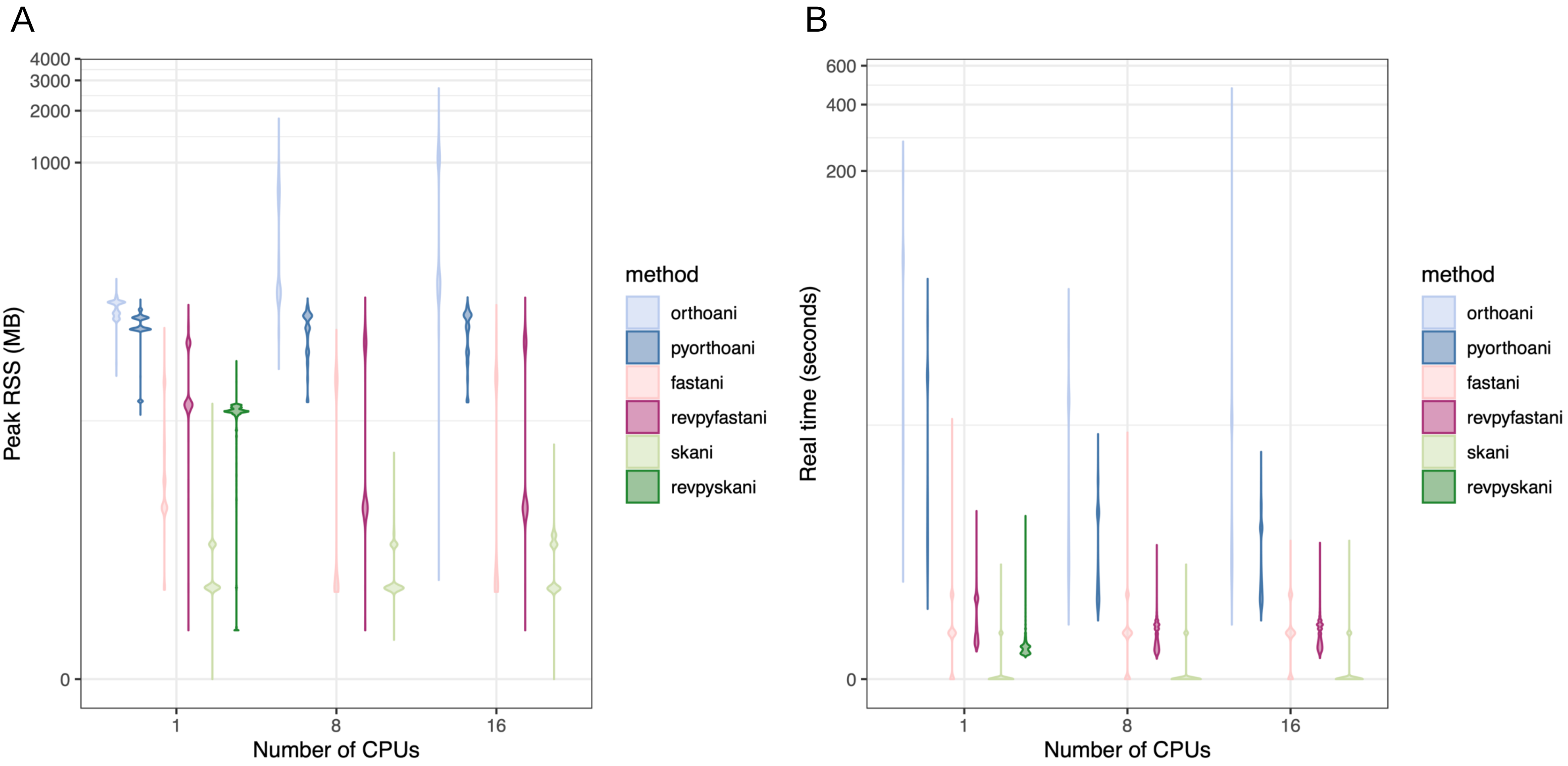

Supplementary Figure S2. Violin plots showing per-genome (A) memory (peak resident set size [RSS], in megabytes [MB]) and (B) time (real/wall clock time, in seconds; Y-axes) used by OrthoANI (orthoani), PyOrthoANI (pyorthoani), FastANI (fastani), PyFastANI (revpyfastani), skani (skani), and Pyskani (revpyskani) on the FastANI validation/benchmarking data sets. For each genome, 1, 8, or 16 CPUs (X-axes) were specified. For Pyskani, only 1 CPU was tested, as Pyskani and skani do not parallelize when performing a single pairwise distance computation (per the skani source code and as demonstrated here).
